# Malignant melanoma with liver and spleen metastases: case report

**DOI:** 10.1590/S1516-31802000000200006

**Published:** 2000-03-02

**Authors:** Laura Cotta Ornellas, Valéria Pereira Lanzoni, Carlos Fischer de Toledo

**Keywords:** Melanoma, Liver metastases, Spleen metastases, Melanoma, Metástases hepática, Metástases esplênica

## Abstract

**CONTEXT:**

The diagnosis of primary melanoma is easily confirmed after histological analysis of the lesion, whereas it is rarely diagnosed when the patient even has distant metastases.

**DESIGN:**

Case report

**CASE REPORT:**

Malignant melanoma is responsible for about 1% of all deaths caused by cancer in the USA and only 3% of all malignant skin diseases. Malignant melanoma is a rare disease, although it corresponds to 65% of all deaths caused by skin cancer. The liver and spleen are rarely the first sites of melanoma metastases. This paper reports on the clinical picture of a patient with fatal malignant melanoma and hepatic and spleen metastases. As this was an unusual presentation, the melanoma diagnosis could only be made after pathological analysis of the skin and hepatic lesions.

## resumo

**CONTEXTO:**

O melanoma maligno é responsável por aproximadamente 1% de todas as mortes causadas por câncer nos EUA e apenas 3% de todas as doenças malignas cutâneas. Embora considerada uma patologia rara, representa 65% de todos os óbitos por câncer de pele. O fígado e o baço raramente são os primeiros sítios de metástases de melanoma.

**TIPO DE ESTUDO:**

Relato de caso.

**RELATO DE CASO:**

Este artigo relata o quadro de um paciente portador de melanoma maligno evoluindo para óbito com metástases hepática e esplênica. Por ser uma apresentação atípica da doença, o diagnóstico definitivo ocorreu somente após a análise histopatológica das lesões de pele e do fígado.

## INTRODUCTION

The diagnosis of primary melanoma is easily confirmed after histological analysis of the lesion, whereas it is rarely diagnosed when the patient even has distant metastases (8% of cases).^1^ This is probably due to the fact that the time between diagnosis of the primary lesion and the appearance of metastases is very long. Klaase et al. (1990) found an average time of 3 years for melanoma and metastases, among 30 patients studied.^2^

Malignant melanoma represents about 1% of all cancers and deaths in the USA but only 3% of all malignant skin diseases, although it is responsible for 65% of deaths caused by skin cancer. It shows a progressive increase in incidence with age, with the patients being mainly between 30 and 60 years old. Melanoma is more common among the white races. The skin pigment has a protective function in colored people, especially the black and yellow races. The most important cause of this cancer seems to be solar exposure.^3^ The primary lesions are located in: limbs (22%), trunk (40%), head and neck (15%), and 16% in unknown sites (4). The most common sites of metastases found in the autopsy are: skin and subcutaneous tissue (75%), lung (70%), liver (68%), small intestine (58%), pancreas (53%), heart (49%), brain (39%) and spleen (36%).^5^ The average survival period for patients with non-visceral metastases is 7.2 months, but it falls to 2.4 months when liver metastases are considered, whether associated with other organs or not.^1^

## CASE REPORT

A 60-year-old white Brazilian male was hospitalized for investigation with a two month history of abdominal pain, altered intestinal function, lack of appetite and asthenia, accompanied by chills and night fever. The patient reported an unquantifiable weight loss and he had been smoking twenty cigarettes a day for the last 30 years.

Physical examination revealed that he had a regular general state, and he was anemic but not jaundiced or febrile. Nodular lesions were observed over the whole body of approximately 1cm diameter, fiberelastic in nature, without infiltration into deeper tissues, covered only by skin of normal aspect. There was one nodule of approximately 2 cm diameter, in the posterior face of the left outer ear that was hardened, ulcerated and associated with nearby angiomas. The liver was observed to be 12 cm below the right costal margin, hardened on its costal edge, nodular, painful, and the Traube space was massive.

Pertinent laboratory findings are shown in Table 1. Biochemical assays showed normal bilirubin, aspartate aminotransaminase (AST) greater than alanine aminotransaminase (ALT), low serum albumin, altered prothrombin activity (PA), high cholestatic enzymes (alkaline phosphatase, AP; and gamma glutamyl transferase, gGT), and very high lactic dehydrogenase (LDH).

**Table 1:** Results of blood examinations at the initial and in the terminal phases of the patient with malignant melanoma

Examinations	Entrance	Terminal phase
Hb/Htc	11.2/33	6.4/19
White blood cells	12200	15500
Differential (leukocytes)	2/70/3/13/11	21/64/0/9/6
Platelets	336000	22700
AST/ALT	55/47	3152/554
AP/gGT	1156/477	1518/348
TB/CB	0.9/0.4	1.1/0.8
PA	57.7%	44.7%
Albumin	2.5	2.1
LDH	5724	12765
Na/K	136/3.9	128/6.0
Hematuria	Negative	320,000
CEA	Normal	
AFP	Normal	
HbsAg	Negative	
HCV antibody	Negative	

Hb- hemoglobin (g/dl); Htc- hematocrit (%); Differential (metamyelocyte, segmented neutrophil, eosinophil, lymphocyte, monocyte); AST- aspartate aminotransaminase (nl: < 37 U/L) and ALT- alanine aminotransaminase (nl: < 40 U/L); AP- alkaline phosphatase (nl: 50-250 U/L) and gGT-gamma glutamyl transferase (nl: 11-43 U/L); TB- total bilirubin and CB- conjugated bilirubin; PA- prothrombin activity; LDH- lactic dehydrogenase; Na- sodium and K- potassium; Hematuria- red blood cells/ml of urine; CEA- carcinoembryonic antigen; AFP- alpha fetus protein; HbsAg- hepatitis B surface antigen.

**Figure 1:**
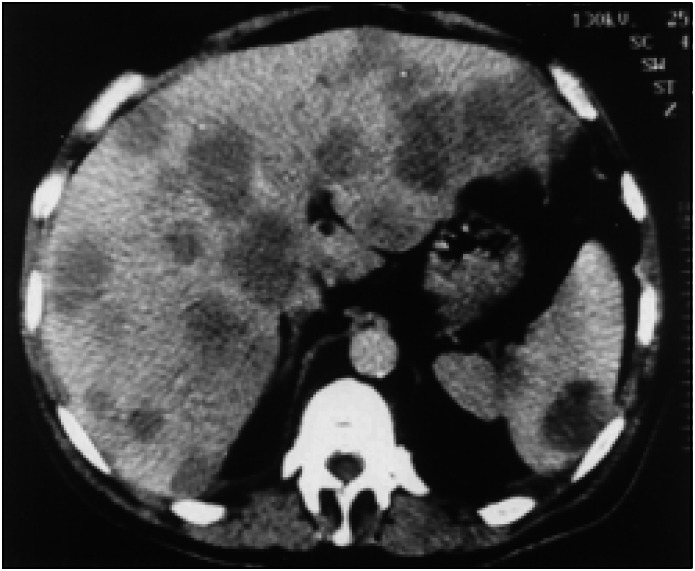
Abdominal computed tomography, showing widespread hypodense nodules in the hepatic and spleen beds, corresponding to a metastatic neoplasm.

**Figure 2:**
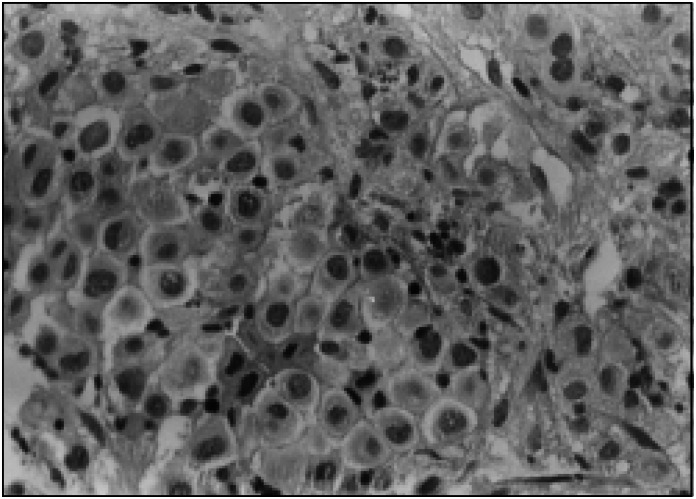
High magnification photomicrograph showing metastatic melanoma in liver tissue. Large cells with vesicular nuclei, prominent nucleoli and eosinophilic cytoplasm (hematoxylin and eosin, 400X).

Oral endoscopy revealed 3 thin varicose cords and scarring from a duodenal ulcer. Sonography of the abdomen showed heterogeneous hepatomegaly and splenomegaly with multiple images suggestive of nodular metastatic lesions in the liver and spleen. There was a nodular image suggestive of peripancreatic ganglia and a left kidney cyst. Computed tomography of the abdomen confirmed the presence of hepatic and spleen nodules, suggestive of metastatic neoplasm (Figure 1). Colonoscopy showed hypertonic diverticular disease. Other exams such as bone scintigraphy with ^99m^Tc-methylene diphosphonate (^99m^Tc-MDP) showed heterogeneous distribution of the radioindicator in the ribs, and computed tomography of the thorax and bronchoscopy did not suggest any malignant lesions. Biopsies (skin and liver) revealed malignant melanoma in the lesion of the left outer ear and melanotic metastases in the subcutaneous tissues and hepatic nodules (Figure 2).

The patient's general state declined rapidly with weight loss, asthenia, painful abdominal distention radiating to the back, ascites, pleural spillage to the right, bilateral edema involving the lower extremities, dyspnea and torpor. After 22 days in hospital, he developed acute anemia and urinary hemorrhage. The final laboratory examinations are shown in Table 1. At this time the patient developed persistent acidosis (pH = 7.25, bicarbonate = 12.1 mmol/L and BE = -12.8) which did not respond to the treatment. Death occurred on the 24^th^ day.

## DISCUSSION

The initial clinical diagnosis was of colorectal cancer with hepatic and splenic metastases, because of the changes in intestinal function and the fact that the large intestine is an usual primary source of liver metastases.^6^ The primary cancer situated on the left outer ear did not have the typical macroscopic characteristics of malignant melanoma. Therefore, the final diagnosis was only conclusive after histological analysis of the lesion.

Despite the low frequency of metastatic tumors in the spleen (4%), these are more frequently encountered in autopsies of patients with melanoma (36%).^5^ The diagnosis of metastatic melanoma in the spleen is rare, although this patient presented splenic metastases at the time of diagnosis. Marked splenomegaly is not common, probably because the average size of the nodules is 1.5 cm.^5^

The frequency with which the liver is observed to be the initial site of melanoma metastases is approximately 4%. The initial clinical presentation includes weakness, anorexia, hepatomegaly and abnormal liver function tests,^5^ which are common in any chronic hepatic disease. The initial laboratory tests (Table 1) showed our case to be chronic hepatic disease, with a cholestasis pattern. There was a significant increase in LDH, suggesting tissue necrosis, which could be described as tumoral invasion.

The initial investigation of abdominal tumor metastases, as for melanoma, should be done using sonography. Computed tomography and nuclear magnetic resonance should also be considered. Melanotic liver metastases are poorly vascularized in hepatic arteriography and small lesions may be easily missed. Diagnostic explorations via radioscintigraphy, especially using ^99m^Tc-DMP, produce both false negative and false positive results and are thus not useful in the initial phases of melanoma when there is no hepatic lesion.^7^ Although the liver biochemical tests showed hepatic alterations, the use of ^99m^Tc-DMP did not appear to add any other conclusion. Hepatic biopsy is a sensitive examination for the diagnosis of metastatic melanoma and the chances of a positive result are increased if the biopsy needle is directed into the lesion under ultrasound, computed tomography or peri- toneoscopic guidance. Das Gupta and Brasfield (1964) showed that the incidence of hepatic metastases at necropsy is 68% and that the nodules are usually multiple, varying in size from 0.5 to 6.0cm diameter, provoking hepatomegaly.^5^

Metastatic melanoma in the skin and/or subcutaneous tissue of the trunk and extremities is found in 75% of patients at autopsy. These metastases result from either lymphatic or vascular dissemination. Melanotic bone metastases are rare (2%) and difficult to determine.^5^

The factors responsible for the death of our patient were probably hemorrhagic and metabolic complications. The destruction of the hepatic parenchyma by the tumor led to significant enzymatic alterations (Table 1), characterized by the increase of AST, ALT and LDH, the cholestatic pattern with high level of gGT and AP, and low serum level of albumin and altered PA. Jaundice usually indicates advanced hepatic disease,^5^ which did not occur in our patient. However the pattern of chronic hepatic disease also contributed to the death of our patient.

At the terminal phase, metabolic acidosis was observed. This could be a consequence of increased production of lactic acid due to inadequate peripheral tissue oxygenation or altered metabolic functions such as insufficient capacity for hepatic clearance of lactate caused by the replacement of normal liver mass by tumor. It is suspected that these metastatic lesions cause local hypermetabolism, thereby worsening hepatic hypoxia and exacerbating lactic acidosis.^8^ Furthermore, the development of ascites and gradual edema of the lower extremities suggests retroperitoneal or mesenteric metastases, which usually have a very bad prognosis.^5^ The patient developed very pronounced anemia due to hematuria exacerbated by low platelets and altered PA. Another contribution to this fatal evolution could have been the infection demonstrated by the left-deviating leukocytosis.

A diagnosis of metastatic melanoma in the bladder can be made in almost all terminal patients with hematuria and melanuria by means of cystoscopy. However, this type of diagnosis is rarely of any practical importance. The main organs of the genitourinary tract that become involved are: kidney (45%), bladder (18%) and prostate (3%).^5^ Hematuria was present in this patient in the terminal stage of the disease and the site of the lesion causing these symptoms was not determined. A necropsy would have been of great diagnostic value for understanding this rapid and fatal evolution. However the patient's family did not authorize this, and it was not performed.

The incidence of and mortality from melanoma are increasing and no effective treatment for the disseminated disease exists. Programs for prevention and early detection of melanoma are therefore warranted.^9^
